# Supplementary material for: Tourist distribution in Northern Mediterranean Basin countries: 2004–2020
Source: PLoS One. 2023 Nov 3;18(11):e0293669. doi: 10.1371/journal.pone.0293669 (PMC10624308; doi:10.1371/journal.pone.0293669)
Supplement: Attachment — Submitted filename: PLOS One Revision_1sept2023.docx [file pone.0293669.s003.docx]

Dear Editor,

Below you can find the rebuttal letter to the reviewer requests regarding our manuscript. Our responses to the comments are indicated in red color below.

**Reviewer #1:**

POINT 1: The authors should include a bibliometric analysis in the title of the paper.

Thank you for your feedback.

The present paper does not only make a bibliometric analysis but rather used a mix methodology including bibliometric analysis and three distinct inequality index, namely; Atkinson, Hoover and Theil index. As such not including *bibliometric analysis* in the title makes more sense.

POINT 2: Abstract: you might try to better clarify the theoretical gap you intend to contribute to. A bit more info could have been added in terms of the used methodology and how do you analyze the data.

To our knowledge, the study as it is the first to analyze the distribution of tourists considering the Mediterranean using the Atkinson, Theil, and Hoover inequality indices and also being supported by bibliometric analysis. This is pointed out in the “originality” section of the abstract

POINT 3: Keywords: The authors should avoid the syntax keywords, for example: ‘’distribution in tourism’’ should be divided to two keywords ‘’Tourism’’, ‘’Distribution’’.

Thank you for the feedback. We have corrected the keywords section accordingly.

POINT 4: A check to eliminate some typos is needed.

Thank you for the comment. The whole text has been proofread again to correct some typos.

POINT 5: The introduction is not solid; it lacks crucial information. An introduction should be insightful and robust, providing readers with a clear understanding of the entire paper.

Thank you for the feedback. In the light of reviewer comments, the introduction part has been substantially expanded. The new and improved paragraphs can be seen with track changes option which are paragraphs # 2,4,5,6,7,8,10,11,12.

POINT 6: It is recommended to state the search structure in the last paragraph of the introduction.

Thank you for the recommendation. The recommended text is added to near the end of the introduction part.

POINT 7: The literature review is not clear.

The manuscript has been substantially expanded to encompass up-to-date literature on the subject.

POINT 8: The authors should justify why choosing WOS rather than Scopus for bibliometric analysis.

WOS was chosen as it is one of the world’s premier databases for published articles and citations and includes publications in top-tier journals and is most suited for literature review (Davaci & Gunkel, 2023). WoS is the most suitable database for data mining and has become one of the primary databases used by scholars for conducting bibliometric analysis (Forliano et al. 2021). Besides, a vast majority of top-tier journals are indexed in WOS and Scopus simultaneously. Therefore, only the WOS database was chosen to conduct the bibliometric analysis of the study.

POINT 9: The authors have neglected many studies conducted in distribution in tourism by using these keywords.

Although there are many studies on tourism using each of inequality indices (Theil, Atkinson and Hoover), there is a shortage of studies measuring inequalities based on the number of tourist arrivals between Mediterranean basin countries. This point is elaborated in the discussion section.

POINT 10: The layout is missed in Figures 2 and 3.

As stated in the PLoS One guidelines, the figures were removed from the manuscript text file and put to the submission as separate files.

POINT 11: The methodology is not well described and justified.

Thank you for the feedback. Based on the recommendation, the methodology is described and justified in the introduction part.

POINT 12: The format of references is not adequate with PLOS ONE requirement.

The references are thoroughly revised to correct any typos and mistakes.

**Reviewer #2: Innovation potential is very limited**

Thank you for the feedback. The novelty of the study is highlighted in the introduction and conclusion parts.

Research hypothesis is not clearly formulated

Thank you for the feedback. Based on the recommendation, research hypothesis is stated in the last part of the introduction

What authors aim to analyze and conclude is not convincingly demonstrated and formulated

The paper, although emphasis in the Tourist sector does not provide and useful information and the state of the art (introduction) does not highlight the importance of hospitality industry. There are too many references highlighting the importance of Mediterranean area and why is chosen from other destinations and this is not explained

In the light of the recommendation, new text has been added that highlight the importance of the hospitality industry and specifically the Mediterranean area along with accompanying references

In addition, methodological approach does not include several elements e.g how the data were collected, how the review was performed, etc. Hence, i couldn't find any statistical analysis or wasn't so clear
Thank you for the recommendation. The requested parts can be seen in the introduction (lines 67-72) as below:

*The number of tourists was analyzed based on the land size of the countries, and the study was repeated using three inequality indices: Atkinson, Hoover, and Theil. The regional distribution of tourists was discussed using data obtained from the World Bank database between 2004 and 2020. The dataset used in this study is derived from the arrivals of tourists in the respective countries.*

results provide typical numbers without any explanation of the importance of the specific KPIs that Authors used.

Thank you for the recommendation. Based on the reviewer’s feedback, explanation of the importance of the KPIs were added to the accompanying section.

The paper lack discussion section in depth

Thank you for the recommendation. In light of the comments, a detailed discussion section is added to the manuscript

**Reviewer #3: The paper topic is interesting. I have some comments, which must be addreesed before the paper is considered for publication.**

1- the authors used WoS and a Turkish database. However, it's worth noting that the adequacy of resources for a bibliometric analysis depends on the specific research question, scope, and objectives of your study. In some cases, researchers may also consider other databases like Scopus, Google Scholar, or specific subject-specific databases to ensure a more comprehensive analysis. It's essential to choose the most appropriate databases and sources based on your research needs and disciplinary context. Please justify why you relied only on these databases.

WOS was chosen as it is one of the world’s premier databases for published articles and citations and includes publications in top-tier journals and is most suited for literature review (Davaci & Gunkel, 2023). WoS is the most suitable database for data mining and has become one of the primary databases used by scholars for conducting bibliometric analysis (Forliano et al. 2021). Besides, a vast majority of top-tier journals are indexed in WOS and Scopus simultaneously. Therefore, only the WOS database was chosen to conduct the bibliometric analysis of the study.

2- the authors mentioned that "This study contributes to the literature by conducting a distribution analysis of annual

tourist gains and analyzing the distribution of incoming tourists based on the country's area

using inequality indices"

However, what does that mean to the existing literature is not discussed.

Thank you for the feedback. Implications of the study on existing literature is elaborated more in the discussion section

3- There are no theoretical or empirical implications sections to discuss what the findings means to the literature or practitioners. How the findings will benefit other than helping in developing global network analysis index.

Thank you for the feedback. In the light of the recommendation, theoretical and managerial implications were added near the end of the conclusion part.

4- in the scope section:

There should be more discussion to the existing literature to know the conceptual background of the topic.

In the light of the recommendation, a number of other related studies were discussed and cited in the introduction and literature review part of the manuscript.

5- There should be either a research context to show data about the northern mideternian countries tourists or include these data in the introduction to make this context familiar to reader from outside the area. Check the below reference for guidance on the research context:

Mahrous, A. A. (2016). Implications of the use of social media for pre-purchase information searches for automobiles. International Journal of Technology Marketing, 11(3), 254-275.

Thank you for the feedback. Based on the recommendation, research context is elaborated in more detail in the introduction part.
